# Electronic Health Record–Based Strategy to Promote Medication Adherence Among Patients With Diabetes: Longitudinal Observational Study

**DOI:** 10.2196/13499

**Published:** 2019-10-21

**Authors:** Stacy Cooper Bailey, Amisha Wallia, Sarah Wright, Guisselle A Wismer, Alexandra C Infanzon, Laura M Curtis, Samantha A Brokenshire, Arlene E Chung, Daniel S Reuland, Allison J Hahr, Kenneth Hornbuckle, Karen Lockwood, Lori Hall, Michael S Wolf

**Affiliations:** 1 Division of General Internal Medicine and Geriatrics Feinberg School of Medicine Northwestern University Chicago, IL United States; 2 Division of Endocrinology, Metabolism, and Molecular Medicine, Department of Medicine Feinberg School of Medicine Northwestern University Chicago, IL United States; 3 Division of Pharmaceutical Outcomes and Policy UNC Eshelman School of Pharmacy University of North Carolina Chapel Hill, NC United States; 4 Division of General Internal Medicine & Clinical Epidemiology Department of Medicine University of North Carolina School of Medicine Chapel Hill, NC United States; 5 Program on Health & Clinical Informatics University of North Carolina School of Medicine Chapel Hill, NC United States; 6 Eli Lilly and Company Indianapolis, IN United States

**Keywords:** health literacy, medication adherence, patient portal, clinical decision support

## Abstract

**Background:**

Poor medication adherence is common; however, few mechanisms exist in clinical practice to monitor how patients take medications in outpatient settings.

**Objective:**

This study aimed to pilot test the Electronic Medication Complete Communication (EMC^2^) strategy, a low-cost, sustainable approach that uses functionalities within the electronic health record to promote outpatient medication adherence and safety.

**Methods:**

The EMC^2^ strategy was implemented in 2 academic practices for 14 higher-risk diabetes medications. The strategy included: (1) clinical decision support alerts to prompt provider counseling on medication risks, (2) low-literacy medication summaries for patients, (3) a portal-based questionnaire to monitor outpatient medication use, and (4) clinical outreach for identified concerns. We recruited adult patients with diabetes who were prescribed a higher-risk diabetes medication. Participants completed baseline and 2-week interviews to assess receipt of, and satisfaction with, intervention components.

**Results:**

A total of 100 patients were enrolled; 90 completed the 2-week interview. Patients were racially diverse, 30.0% (30/100) had a high school education or less, and 40.0% (40/100) had limited literacy skills. About a quarter (28/100) did not have a portal account; socioeconomic disparities were noted in account ownership by income and education. Among patients with a portal account, 58% (42/72) completed the questionnaire; 21 of the 42 patients reported concerns warranting clinical follow-up. Of these, 17 were contacted by the clinic or had their issue resolved within 24 hours. Most patients (33/38, 89%) who completed the portal questionnaire and follow-up interview reported high levels of satisfaction (score of 8 or greater on a scale of 1-10).

**Conclusions:**

Findings suggest that the EMC^2^ strategy can be reliably implemented and delivered to patients, with high levels of satisfaction. Disparities in portal use may restrict intervention reach. Although the EMC^2^ strategy can be implemented with minimal impact on clinic workflow, future trials are needed to evaluate its effectiveness to promote adherence and safety.

## Introduction

### Background

Medication nonadherence has long been recognized as a major public health and patient safety concern, costing the US health care system billions annually and compromising the benefit and risk profile of patients’ treatments [1,2]. Nonadherence has been linked to negative health outcomes across a number of chronic conditions, including poorer disease control, increased hospitalizations, and greater morbidity and mortality [3-6]. However, decades of research has shown that poor medication adherence in its many forms—failure to fill, early abandonment of therapy, and discontinuation—continues to be common and pervasive [3,7-9].

Despite its prevalence and consequences, medication adherence is often inadequately addressed during clinical encounters. A number of studies have shown that provider-patient communication on medication use is suboptimal [10-13]. As a result, patients are often poorly informed about treatment plans and lack the knowledge necessary to manage medications safely and effectively on their own [9,14]. From the provider perspective, it is difficult to objectively, efficiently, and accurately collect information on patients’ medication adherence in time-constrained clinical settings [13]. Information that is obtained may not be clinically actionable, and few programs have been widely implemented to monitor outpatient medication use and intervene when necessary should safety or adherence concerns be detected [9].

### Technology-Based Solutions

Recently, increased attention has been placed on how health information technology can be used to collect data on patient medication use with the goal of improving outpatient medication adherence and safety [9,15-17]. The Health Information Technology for Economic and Clinical Health Act and the Office of the National Coordinator’s Meaningful Use initiative led to a dramatic increase in the adoption of electronic health records (EHRs) in health care systems nationwide [18]. In the context of outpatient medication use, EHRs can provide a unique opportunity to improve medication monitoring and safety, particularly via their associated tethered patient portals, which can better connect patients to their providers in outpatient settings. This is a distinct advantage of EHR and tethered portal-based interventions in comparison with other consumer technologies (eg, short message service (SMS) text messaging and mobile apps), which are often not as easily linked and integrated with clinical care workflows or clinical data.

Although EHRs and tethered portals have tremendous potential, there have been a limited number of methodologically rigorous studies that have sought to develop and evaluate EHR-based interventions to promote and monitor safe outpatient medication use. Graumlich et al evaluated MedTable, a patient education tool embedded within the EHR, which promoted medication review and reconciliation between patients and providers in a randomized controlled trial among 674 patients with type 2 diabetes [19]. They found that intervention patients had greater knowledge of medication indications but did not experience improved medication adherence or hemoglobin A_1c_. Similarly, both Weingart et al and Schnipper et al developed and evaluated EHR modules that promoted medication review and reconciliation by patients and providers in 2 randomized controlled trials [20,21]. Although Weingart et al found that intervention patients in their trial had more accurate medication lists with fewer discrepancies with the potential for severe harm, neither study reported a reduction in adverse drug events among intervention patients [20,21].

In terms of medication adherence, Volmer et al found that a multifaceted intervention, which included an EHR-based component, significantly increased adherence to statins and angiotensin-converting enzyme inhibitors/angiotensin receptor blockers among intervention patients compared with usual care; however, their strategy utilized automated telephone reminders, personalized letters, and live outreach, not solely EHR functions [16]. Similarly, Simon et al investigated the effectiveness of a depression care management program delivered via messaging through an EHR [22]. They found that intervention patients had higher antidepressant adherence rates and fewer depression symptoms at 5 months than usual care patients. However, this trial was conducted in a large integrated health care system with greater care coordination between primary care and mental health providers; authors acknowledged that the intervention strategy may not be feasible in other practice settings.

### Study Purpose

Although EHRs and their associated portals offer a unique opportunity to bridge patients to their providers, prior studies have reported variable success in using EHR-based strategies to support and monitor medication use in outpatient settings [16,19-22]. Those interventions that appear to have been the most successful to date may be less feasible for widespread implementation. To address this concern, our team recently devised and pilot-tested the Electronic Medication Complete Communication (EMC^2^) strategy, a simple, sustainable approach that uses EHR technology to promote medication adherence and safety by providing routine assessment and monitoring of patients’ medication use at home. Herein, we describe the core components of the EMC^2^ strategy and results from our initial pilot test, which focused on building the technological infrastructure within the EHR and portal to support the intervention as well as determining the feasibility of the strategy and its acceptability among a diverse set of patients from both primary care and specialty practices.

## Methods

### Overview

The EMC^2^ strategy is a multifaceted intervention that consists of several components designed to (1) promote provider counseling on medication adherence and safe use, (2) provide patient-friendly education at the point of prescribing, (3) monitor patient medication use in outpatient settings, and (4) prompt clinic follow-up with patients reporting medication-related concerns. We used the Institute of Medicine’s *Medication Use Process Model* to inform our strategy and the above components; this model deconstructs common system failures in the processes of prescribing, dispensing, self-administering, and monitoring medications that commonly lead to medication errors, adverse drug events, and suboptimal adherence and treatment [23].

The EMC^2^ strategy is intended to be flexible, with intervention components that can be adapted for specific settings and patient populations. Concurrent to this project, other iterations of the EMC^2^ strategy have been developed to provide monitoring via interactive voice recognition technology among low-income, primary care patients and to monitor opioid use among emergency department patients [24,25]. For this study, the EMC^2^ strategy was delivered via Epic EHR Software (Epic Systems Corporation) at both study sides. The functionalities described are not specific to Epic, however, and could be deployed in other EHRs with tethered patient portals. Our team has implemented EHR-based interventions using similar functionalities across a number of distinct EHR platforms [25,26].

### Study Medications

For the purpose of this study, we tailored the EMC^2^ strategy to support medication adherence and safe use among patients with diabetes, a chronic condition that is becoming increasingly prevalent and for which medication use is a cornerstone of self-management [3,27]. We selected various prescription medications used to treat diabetes and their associated generic formulations as targets for the intervention. These medications were selected as most are considered higher risk according to the Food and Drug Administration (FDA) and consequently require an FDA-approved Medication Guide. Despite these regulations, patients may not receive adequate counseling or monitoring on higher-risk medications during and after the clinical encounter [10,23].

### Intervention Components

The EMC^2^ strategy tested in this study included 4 core components, as described below.

#### Best Practice Alert for Physicians

Best practice alerts (BPAs) are clinical decision support alerts within Epic EHR systems that provide a mechanism to show relevant data in a pop-up window at the point-of-care. When a prescriber placed a new order or refill for 1 of the study medications, a BPA fired to prompt the prescriber to counsel the patient on medication use and key medication risks. The BPA also listed key adverse events associated with the medication according to the FDA Medication Guide and/or prescriber insert and included a hyperlink within the BPA window to additional medication information.

#### Automated Delivery of Food and Drug Administration Medication Guide + Medication Summary

Upon placing an order for a higher-risk medication, an FDA Medication Guide and a 1-page, patient-friendly *Medication Summary* were automatically queued for printing along with the patients’ after-visit summary (AVS). The AVS is a handout that provides the patient with their care plans, medication lists, and other information that is provided at the conclusion of the clinical encounter. The Medication Summary provided an overview of medication instructions, risks, and benefits in patient-friendly language. Medication Summaries were previously developed by our research team and have been shown to support patient comprehension of medication information [28].

#### Follow-Up Portal Assessment

Approximately 1 week after their clinic visit, patients with a portal account were sent an email prompting them to log on to the portal to complete and submit a brief, voluntary questionnaire. This questionnaire consisted of 9 questions, which were developed by our study team, to assess common root causes identified in the scientific literature as being related to medication adherence and safety (ie, forgetfulness, cost, side effects; see Multimedia Appendix 1) [29]. The online survey was only available by invitation to those who had enrolled in the study and had a portal account. All items on the survey appeared on 1 page and could be easily completed by patients. Patients were able to review responses to their questions before submission but were only allowed to complete the questionnaire once. Patients without a portal account did not receive any further contact.

#### Clinic Follow-Up

The results of the patient portal questionnaire were automatically sent via an EHR message to clinic staff. Clinic staff reviewed results then followed up with any identified medication-related concerns. Clinics were asked to determine their own specific protocols for follow-up; these were separate from research activities.

### Study Design

To determine the feasibility and acceptability of the EMC^2^ approach, a single-arm study was conducted to build and pilot-test the EMC^2^ strategy in 2 academic health care settings: an endocrinology clinic in Chicago, Illinois, and a general internal medicine clinic in Chapel Hill, North Carolina. Both clinics utilized Epic EHR systems.

The EHR build included programming the BPAs, Medication Summaries, and FDA Medication Guides to trigger and launch whenever a medication order was placed for 1 of the study medications. This functionality was delivered to all patients who visited a study clinic and received a prescription for 1 of the study medications, regardless of their study involvement; this ensured that patients received all in-clinic EMC^2^ components on the day of their index clinic visit. Only those patients who consented to participate in the study following their clinic appointment were eligible to receive the remaining EMC^2^ strategy components (ie, portal assessment and clinic follow-up) and to participate in evaluation activities. The institutional review boards of Northwestern University and the University of North Carolina at Chapel Hill approved study procedures.

### Participants

Patients were considered eligible for the study if they (1) were aged 18 years or older, (2) spoke English, (3) were primarily responsible for administering their own medication, (4) received a new or refill prescription for a study medication, and (5) had access to the internet. Adults with severe, uncorrectable vision, hearing, or cognitive impairments, or who were unable to provide informed consent, were excluded from participating. Adults without a portal account were allowed to enroll in the study and were told that they could sign up for an account if they desired.

To recruit patients for the evaluation of the EMC^2^ strategy, EHRs were configured with eligibility criteria such that a message was sent to research assistants whenever a study medication was ordered for a patient in the study clinic. Research assistants then contacted identified patients by either approaching them following their clinic visit or calling them via telephone following their appointment. Research assistants then introduced the study, verified eligibility, and enrolled interested and eligible patients in the study. Participants completed a baseline, in-person interview then a telephone interview 2 weeks post clinic visit. Data were collected using REDCap software (Research Electronic Data Capture) hosted by Northwestern University [30]. Participants were compensated for participating in study interviews; the same incentive was received whether participants completed the follow-up portal assessment or not.

### Measurement

#### Participant Characteristics

Patient sociodemographic variables (eg, race, income, age, and sex) were collected during the baseline interview. Patient health literacy skills were measured via the Newest Vital Sign (NVS), a commonly used assessment that asks patients to interpret information presented on a standard Nutrition Facts label [31]. Information regarding patients’ prior use of technology and the patient portal was also collected based on patient self-report.

#### Process Outcomes

Outcomes related to the fidelity of the intervention were collected via patient self-report and EHR data. Specifically, during the baseline interview, patients were asked to self-report receipt of provider counseling during their index clinic visit (yes/no) and receipt of a Medication Summary following their clinic visit (yes/no). Data from the EHR, collected after the 2-week interview, were used to determine if patients received and completed the portal questionnaire and to determine if any clinic follow-up occurred based upon responses to the portal survey.

#### Patient Satisfaction

Patient satisfaction with the various EMC^2^ strategy components was also assessed. During the baseline interview, patients were asked about communication with their providers on medication use via a modified version of the supplemental Health Literacy items from the Consumer Assessment of Healthcare Providers and Systems questionnaire [18]. They were also asked to rate their satisfaction with the Medication Summary and their perceived helpfulness of the tool on a scale of 1 to 10. During the 2-week interview, patients were asked about their continued satisfaction with the Medication Summary and whether it was still in use (yes/no). Finally, during the 2-week interview, patients reported on their experiences with the portal questionnaire, including any barriers to its completion (eg, difficulty logging on to the portal and challenges understanding or answering questions), and their overall satisfaction with the tool (scale of 1-10).

### Statistical Analysis

Descriptive statistics were calculated for patient sociodemographic variables, process outcomes, and variables related to patient satisfaction with the intervention. To assess whether there were any systematic, statistically significant differences between patients who had access to the portal and those who did not, we used Pearson chi-squared tests or Fisher exact tests for categorical variables and Student *t* tests for age. The same tests were used to compare the differences between the patients who completed the portal questionnaire versus those who did not in those with portal access. Specifically, we examined if there were variations by age, sex, race, education, income, health literacy skills, and study site. Statistical significance was defined as alpha<.05.

## Results

### Overview

Recruitment began in December 2016 and concluded in April 2017. A total of 251 patients were approached or contacted by a research assistant; 66 patients declined to participate, and 31 patients were ineligible. A total of 100 patients completed the baseline interview (n=43 in North Carolina, n=57 in Illinois), and 90 patients completed the 2-week follow-up interview. There were no significant differences in sociodemographic characteristics between those who completed the follow-up interview and those who did not.

### Participant Characteristics

Table 1 describes the characteristics of the baseline study sample (N=100). The mean age was 56.2 (SD 11.20; range 24-82) years. More than half (57/100, 57.0%) of the sample was female, about one-third were African American (38/100, 38.0%), and 30.0% (30/100) reported a high school or less level of education. A total of 40 patients (40/100) had limited health literacy skills (low or marginal) according to the NVS. About a quarter (28/100, 28.0%) of patients reported not having a portal account. Among those who reported having and using a portal account (n=67), 54% (36/67) stated that they used the portal once per month or less. Disparities were noted in portal account ownership by level of education, household income, race, and study site (Table 1).

### Process Outcomes

Almost two-thirds (63/100, 63.0%) of patients reported that their physician counseled them on how to take their medication, and 54.0% of patients (54/100) stated that their physician counseled them on possible side effects of the medication. The majority of patients (72/100, 72.0%) reported receiving a Medication Summary after their index clinic visit. Of those who completed the 2-week follow-up interview, 74% (67/90) reported that they still had the Medication Summary in their possession.

**Table 1:** Participant characteristics, portal account ownership, and questionnaire completion.

Participant Characteristic			All participants (n=100)	Portal account		Portal questionnaire	
				Proportion with account (n=100)	*P* value	Proportion completed (n=72)	*P* value
**Overall, n (%)**			—^a^	72 (72.0)	—	42 (58)	—
	**Age (years)**		—	—	.09	—	.50
		<55	42 (42.0)	35 (83.3)	—	22 (63)	—
		55-64	34 (34.0)	21 (61.8)	—	10 (48)	—
		>65	24 (24.0)	16 (66.7)	—	10 (63)	—
	**Sex**		—	—	.36	—	.28
		Male	43 (43.0)	33 (76.7)	—	17 (52)	—
		Female	57 (57.0)	39 (68.4)		25 (64)	
	**Race^b^**		—	—	<.01	—	.70
		Black	38 (38.0)	20 (52.6)	—	10 (50)	—
		White	48 (48.0)	39 (81.3)	—	24 (62)	—
		Other	13 (13.0)	12 (92.3)	—	7 (58)	—
	**Education**		—	—	.02	—	.01
		High school or less	30 (30.0)	16 (53.3)	—	4 (25)	—
		Some college	33 (33.0)	25 (75.8)	—	16 (64)	—
		College graduate	37 (37.0)	31 (83.8)	—	22 (71)	—
	**Income^c^** **(US $)**		—	—	.03	—	.07
		<30,000	24 (24.0)	13 (54.2)	—	4 (31)	—
		30-49,999	20 (20.0)	15 (75.0)	—	9 (60)	—
		50-99,999	27 (27.0)	21 (77.8)	—	13 (62)	—
		>100,000	23 (23.0)	21 (91.3)	—	16 (76)	—
	**Literacy**		—	—	.10	—	.12
		Low	12 (12.0)	6 (50.0)	—	1 (17)	—
		Marginal	28 (28.0)	19 (67.9)	—	11 (58)	—
		Adequate	60 (60.0)	47 (78.3)	—	30 (64)	—
	**Site**		—	—	<.01	—	.09
		Chicago	57 (57.0)	51 (89.5)	—	33 (65)	—
		North Carolina	43 (43.0)	21 (48.8)	—	9 (43)	—

^a^Not applicable.

^b^n=1 refused race.

^c^n=6 do not know or refused income.

Among patients with a portal account, 58% (42/72) completed the portal questionnaire. Patients with lower levels of education were less likely to complete the questionnaire (*P*=.01). Among those patients who submitted a questionnaire (n=42), 12 did not report any medication-related concerns on the survey, 10 reported minor concerns that did not warrant follow-up according to individualized clinic protocols, and 21 identified issues that triggered a clinical response. Of the latter, 81% (17/21) were contacted by the clinic or had their medication-related issue resolved within 24 hours. The remaining 4 patients received clinic outreach within 5 days. The most frequent problems identified in the portal questionnaire were not currently having the medication in possession (n=34), difficulties paying for the medication (n=5), and worries about side effects (n=17).

### Patient Satisfaction

Overall, patients reported high levels of satisfaction with communication with their providers on medication use and with the intervention components. Among patients who reported receiving counseling on how to take their medications, 97% (61/63) said the guidance was very or mostly easy to understand. Similarly, among those adults reporting receiving counseling on side effects (n=54), all said the counseling was very or mostly easy to understand.

Almost all (93/96, 97%) of the patients felt the Medication Summaries were very easy or mostly easy to understand. On a scale from 1 to 10 (with 10 being the best), patients scored the Medication Summary an average of 9.0 (SD 1.6) in being clear and 9.2 (SD 1.3) in being helpful. All of the 38 patients who completed both the portal questionnaire and the follow-up interview reported that the portal-based questionnaire was very easy or somewhat easy to access. A total of 89% (33/38) of patients completing the portal questionnaire and the follow-up interview reported high levels of satisfaction with the portal experience (8 or more on a scale of 1-10).

## Discussion

### Principal Findings

Results from this study indicate that the EMC^2^ strategy can be reliably implemented and delivered to patients. Medication Summaries were received by the majority of patients at their index clinic visit, and most patients still had the materials in their possession 2 weeks after their clinic visit. Most patients who had a patient portal account completed the online questionnaire, and clinic staff were able to resolve issues identified in the questionnaires in a timely manner. Importantly, patients reported high levels of satisfaction with intervention components, and the EMC^2^ strategy appears to have been successfully implemented in 2 diverse practices with minimal impact on clinic workflow.

Although there were many positive results from this feasibility study, we also uncovered some shortcomings to the EMC^2^ strategy. More than a quarter of patients enrolled in the study did not have access to the patient portal and were, therefore, unable to complete the questionnaire. Significant socioeconomic disparities were found between those patients who had portal access and those who did not. These findings are consistent with prior studies and likely mirror socioeconomic disparities in the internet and portal access and use [32,33]. Similarly, we also found disparities in questionnaire completion among patients who had portal access. Specifically, patients with less educational attainment were less likely to complete the portal questionnaire, even though they had portal access. It is possible that patients with lower education levels may need additional technical assistance and support to effectively utilize the patient portal and to complete portal-based questionnaires. It is possible that follow-up assessments may need to be conducted via telephone or another modality for a limited number of patients, with results recorded in the EHR for the care team to review and address.

Although these disparities limit the current reach of the EMC^2^ strategy, data indicates that internet access and use is on the rise and that US adults are becoming increasingly familiar with using technology to support their health [34,35]. Thus, although the EMC^2^ strategy may not work for all patients at this point in time, it is plausible that its reach will increase greatly in years to come. This is also likely given the large investments made in health information technology by health care systems nationwide and the increasing attention placed on promoting the use of patient portals by health systems.

### Limitations

There are limitations to this study that should be noted. Specifically, this was a small pilot study that was focused on building the EMC^2^ strategy components in the EHR and portal and determining the feasibility of implementing the strategy in 2 diverse clinics within 2 separate health systems. As such, our focus was on understanding the delivery of the EMC^2^ strategy and patient satisfaction with the intervention components; additional studies are needed to fully evaluate the effectiveness of the strategy itself. Although participants were recruited from both specialty and primary care practices in 2 diverse locations, we only included English-speaking patients in our study, which limits study generalizability. Future work is needed to develop intervention materials and to test the strategy among non-English speaking populations.

### Conclusions

Physician time for counseling and monitoring medication use in outpatient settings is extremely limited. Studies have shown that providers would need to spend approximately 18 hours per working day to deliver counseling and care consistent with US Preventive Services Task Force and chronic conditions care management recommendations [36,37]. To improve quality of communication around medication adherence and safety, hard-wired approaches such as the EMC^2^ strategy are, therefore, clearly needed. EMC^2^ tools could help streamline physician counseling, and written materials could support patient learning on safe and appropriate medication use outside of the clinic visit. Utilizing the patient portal to provide outpatient monitoring of medication use can help identify those patients in greatest need of additional follow-up. Importantly, the strategy can also help patients engage with other members of the care team (ie, nurses and care coordinators) so that counseling is not entirely dependent upon physicians during time-limited visits. Additional research is currently underway utilizing a rigorous, randomized study design to formally test the effectiveness of the EMC^2^ strategy against usual care.

## Appendix

Multimedia Appendix 1 Portal questionnaire.

## Abbreviations

AVS: after-visit summary
BPA: best practice alert
EHR: electronic health record
EMC2: electronic medication complete communication
FDA: Food and Drug Administration
NVS: Newest Vital Sign
