# Supplementary material for: Electronic Health Record–Based Strategy to Promote Medication Adherence Among Patients With Diabetes: Longitudinal Observational Study
Source: J Med Internet Res. 2019 Oct 21;21(10):e13499. doi: 10.2196/13499 (PMC6829279; doi:10.2196/13499)
Supplement: Multimedia Appendix 1 [file jmir_v21i9e13499_app1.pdf]

Multimedia Appendix. Portal Questionnaire

We would like to ask you a few questions about your [Medicine name].
Please answer each question as best as you can. We appreciate your honesty.

1. During the past week, did you have any of this medicine available for you to take?

   Yes *1*

   No...*0*

   If no, please go to the end of the survey and click on the submit button.

2. During the past week, did you decide to stop taking this medicine without telling your doctor?

   Yes *1*

   No...*0*

3. During the past week, did you decide to take more or less of this medicine than you were supposed to without telling your doctor?

   Yes *1*

   No...*0*

4. Did you forget to take any of this medicine yesterday?

   Yes *1*

   No...*0*

5. Did you forget to take any of this medicine the day before yesterday?

   Yes *1*

   No...*0*

6. Did you forget to take any of this medicine 3 days ago?

   Yes *1*

   No...*0*

7. Are the instructions on how to take medicine confusing to you?

Yes..*1*

No...*0*

8. Did you have a hard time paying for this medicine the last time you bought it?

Yes..*1*

No...*0*

9. Do you worry about this medicine causing side effects?

Yes..*1*

No...*0*

If you think you are having a serious side effect from your medicine, you should contact your doctor immediately.

[Medicine name] may cause low blood sugar or hypoglycemia. Some signs of low blood sugar are:

- having a very bad headache
- feeling unusually drowsy, weak or dizzy
- feeling anxious or unable to sit still
- blurry vision
- sweating more than usual
- confusion
- irritability
- feeling hungrier than usual
- fast heartbeat

You should talk to your doctor if you have any questions or concerns about your medicine.

Please click on the submit button below. Thank you for answering our questions.
